# Editorial: Frailty in older patients during the COVID-19 era

**DOI:** 10.3389/fmed.2023.1348468

**Published:** 2024-01-05

**Authors:** Claudio Tana

**Affiliations:** Geriatrics Clinic, SS. Annunziata Hospital of Chieti, Chieti, Italy

**Keywords:** frailty, COVID-19, SARS-CoV-2, Long COVID, elderly

Frailty is a complex syndrome characterized by an impairment in activities of daily living (ADL) and a reduced physiological reserve, which is associated with a high vulnerability to stressor events (1).

Frailty is a common condition in older adults but can affect any age group depending on risk factors. Prolonged bed rest, the presence of comorbidities, and malnutrition can occur at any age and increase the risk of complications (2). The recent pandemic of novel coronavirus disease 2019 (COVID-19), especially in the first wave of the pandemic, was associated with a significant risk of mortality in almost all age groups who exhibited specific risk factors (3, 4). Lung damage and acute cardiovascular disease were the major causes of mortality in patients with COVID-19 (5, 6). An increase in the burden of frailty and mortality from COVID-19 has been documented in frail older adults, who have an increased risk of morbidity and mortality from COVID-19 when compared to those without this syndrome (7). The immunopathogenesis is still largely unknown, but it has been hypothesized that dysregulation of the immune system, inflammation, and immunosenescence seem to play a pivotal role (8). Long COVID symptoms in patients who have survived the acute infection are another emerging issue, and older adults may be particularly affected (9). Gut dysbiosis also appears to contribute to immune dysregulation and disease severity. Indeed, it has been hypothesized that an increase in opportunistic bacteria during SARS-CoV-2 infection, along with age-related alterations in the intestinal microbiota, is associated with muscle loss, insulin resistance, high oxidative stress, and systemic inflammation (10). The epidemiology of COVID-19 in older adults, clinical characteristics, and age-related mechanisms of disease are the subject of this Research Topic.

Fernandes and Pereira reported that frail older adults affected by COVID-19 account for ~51% of hospitalized patients and have a high risk of in-hospital death that correlates with the synergistic effect of COVID-19 severity and age-related frailty. The authors summarized the results of meta-analyses of the impact of frailty on short-term mortality in hospitalized older adults with COVID-19.

In their paper, Kashtanova et al. conducted a multi-level evaluation based on a comprehensive geriatric assessment of major age-associated risk factors and found that the most significant predictors of mortality among post-COVID-19 participants were depression, frailty, and frontal lobe dysfunction, and blood markers such as low levels of IGF-1, HDL, and 25-hydroxyvitamin D and high levels of pro-inflammatory markers and white blood cells were all associated with a worse outcome. Length of hospital stay and frailty index were factors that significantly increased the risk of mortality in the study by Vainqueur et al..

Therefore, COVID-19 remains a significant challenge for older adults. Regular vaccination campaigns and preventive measures aimed at reducing SARS-CoV-2 infection are particularly encouraged, especially among older adults, who remain the main age group at risk for this disease.

## Author contributions

CT: Conceptualization, Writing—original draft, Writing—review & editing.
